# Evaluation of antiviral activity of *Carica papaya* leaves against SARS-CoV-2 assisted by metabolomic profiling

**DOI:** 10.1039/d2ra04600h

**Published:** 2022-11-16

**Authors:** Amr Adel, Mohamed S. Elnaggar, Amgad Albohy, Ahmed A. Elrashedy, Ahmed Mostafa, Omnia Kutkat, Usama Ramadan Abdelmohsen, Eman Al-Sayed, Mohamed A. Rabeh

**Affiliations:** Department of Pharmacognosy, Faculty of Pharmacy, Modern University for Technology and Information 11865 Cairo Egypt; Department of Pharmacognosy, Faculty of Pharmacy, Ain Shams University 11566 Cairo Egypt; Department of Pharmaceutical Chemistry, Faculty of Pharmacy, The British University in Egypt (BUE) Suez Desert Road ElSherouk City 11837 Cairo Egypt; Natural and Microbial Chemistry Department, Pharmaceutical and Drug Industries Research Division, National Research Centre (NRC) AlBohoos Street, Dokki 12311 Cairo Egypt; Center of Scientific Excellence for Influenza Viruses, National Research Centre 12622 Giza Egypt; Department of Pharmacognosy, Faculty of Pharmacy, Minia University Minia 61519 Egypt; Department of Pharmacognosy, Faculty of Pharmacy, Deraya University Minia 61111 Egypt; Department of Pharmacognosy, Faculty of Pharmacy, Cairo University 11562 Giza Egypt

## Abstract

The COVID-19 pandemic caused a huge health crisis all over the globe. SARS-CoV-2 is the virus responsible for the disease and it is highly contagious leaving millions of confirmed infected cases and a dangerous death toll. *Carica papaya* is a tropical plant known for its antiviral activity since it possesses different classes of compounds that are believed to combat various viral classes. In this study, the extracts prepared from *C. papaya* leaves cultivated in Egypt were evaluated for their anti-SARS-CoV-2 activity using crystal violet assay and for their cytotoxicity through MTT assay. The total methanolic extract, *n*-hexane, ethyl acetate, and *n*-butanol fractions of papaya leaves were used in the study and the results revealed that the *n*-hexane fraction has a high anti-SARS-CoV-2 activity with an IC_50_ value = 1.98 μg mL^−1^. Moreover, it also showed a high selectivity index value = 104.7. Dereplication of the secondary metabolites in the crude methanolic extract of *C. papaya* leaves revealed the presence of different classes of compounds including sterols, terpenes, fatty acid, alkaloids and flavonoids that are known to possess antiviral activities against various classes of viruses. The current study was assisted by molecular docking, molecular dynamics simulation and MM-PBSA calculations for the annotated compounds against 6 SARS-CoV-2 target proteins. The results of these *in silico*-based investigations showed high to moderate binding on the targeted proteins. This postulation may instigate further research studies concerning the compounds responsible for this high anti-SARS-CoV-2 activity of the *n*-hexane fraction of *C. papaya* leaves.

## Abstract 2

The *n*-hexane extract showed a significant antiviral activity. In addition, annotated compounds exhibited high binding scores on 6 target viral proteins *in silico*.

## Graphical abstract

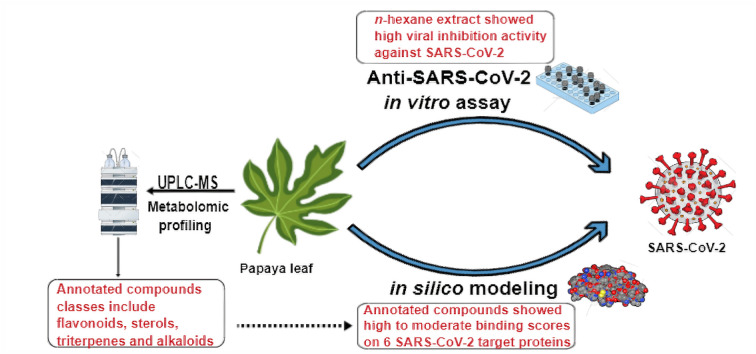

## 1. Introduction

Severe acute respiratory syndrome coronavirus (SARS-CoV) and Middle East respiratory syndrome coronavirus (MERS-CoV) caused severe acute respiratory diseases in the last two decades through transmission from animals to humans.^1^ But in December 2019, a novel severe acute respiratory syndrome coronavirus (SARS-CoV-2) inflicted a disturbance to the global health care and economy systems and affected the lives and health of millions all over the world.

Herbal medicines are considered a rich source of natural antiviral compounds that proved their activity against different classes of viruses. Therefore, natural compounds may help in the combat against SARS-CoV-2. *C. papaya* is an edible tropical fruit native to Central America and Mexico.^2^ Papaya leaves, seeds, roots, and fruits have been widely used in folk medicine in Africa and Central America in the treatment of various diseases such as dengue fever, jaundice, asthma, gonorrhea, urinary complaints, dressing wounds. Also, in the Ayurvedic literature, it was used as a laxative, diuretic, expectorant, antibacterial, antifungal, counter-irritant and as a treatment for dysentery and chronic diarrhea, also as a paste for treatment of ringworm and psoriasis.^3,4^ Many pharmacological activities were reported for *C. papaya* including anti-inflammatory,^5^ anti-cancer,^6^ anti-protozoal,^7^ anti-microbial,^8^ anti-diabetic,^9^ anti-fungal,^10^ anti-hyperlipidemic,^11^ anti-thrombocytopenic,^12^ anti-viral,^13^ anti-gout,^14^ antihypertensive,^15^ analgesic^16^ and hepato-protective activities.^17^ The wide range of activity is due to the presence of different secondary metabolites including flavonoids, alkaloids, sterols, triterpenoids, isothiocyanates, tannins, and other phenolic compounds.

The different extracts of *C. papaya* possessed previously reported antiviral activities. For example, it is well known for its activity in the treatment of the dengue virus.^18–21^ In addition, papaya fruit pulp was recently reported to exert antiviral activity against Zika virus.^22^ Papaya comprises a wide range of phyto-compounds that may induce antiviral activities against different classes of viruses.^23^ Fatty acids, sterols, and triterpenoid compounds represent the major compounds present in *C. papaya*.^24–26^ Many studies reported that fatty acids mediate antiviral activities *via* several mechanisms.^27–29^ Moreover, sterols and triterpenoids had significant antiviral activity against different viral types.^30,31^ Betulinic acid, oleanolic acid, and stigmasterols were from the major triterpenoid and steroidal compounds present in papaya with reported antiviral activities. Betulinic acid, a pentacyclic triterpene, displayed a broad range of antiviral activities against human immunodeficiency virus (HIV) and herpes simplex virus (HSV).^32^ In addition, it showed activity against Influenza A and ECHO 6 viruses.^33^ Another pentacyclic triterpene oleanolic acid demonstrated antiviral activities against HIV-1 and Hepatitis C virus (HCV).^34–36^ Furthermore, stigmasterol is one of the famous steroidal compounds reported in papaya.^37^ This sterol is known to have inhibitory activity against the HIV-1 virus's reverse transcriptase.^38^ These significant antiviral activities initiated the idea of testing the possible antiviral activity of different extracts of the leaves of *C. papaya* cultivated in Egypt that could contain promising antiviral agents against SARS-CoV-2.

## 2. Experimental

### 2.1. Plant material

The plant material of *C. papaya* leaves was collected in August 2018 from the home garden, Nasr city, Cairo, and was authenticated by Eng. Therease Labib, the consultant at Orman Botanical Garden, Giza, and National Gene Bank at the Ministry of Agriculture, Egypt. A voucher specimen of *C. papaya* (PHG-P-CP-382) was deposited at the herbarium of the Department of Pharmacognosy, Faculty of Pharmacy, Ain Shams University, Cairo, Egypt. The collected leaves were washed with tap water to remove any dust and contaminants and shade dried for 5 days till complete dryness. The dried leaves were then collected and milled to a fine powder to be ready for extraction.

### 2.2. Chemicals

All the reagents used were of an analytical grade. The chemicals are purchased from Piochem Company and Al-Nasr Company for chemical industries.

### 2.3. Extraction procedure

The powdered leaves of *C. papaya* (1.7 kg) were extracted by three times maceration with a total of 17 L of methanol at room temperature for 24 h. The extract was then filtered, stored, and protected from sunlight to be evaporated. The crude extract was then evaporated at 50 °C under reduced pressure using a high-capacity rotary evaporator at the research lab unit of the faculty of pharmacy, Ain Shams University. A total dark green crude extract with a weight of (245 g) was produced, collected in a sterile flask for further fractionation.

### 2.4. Fractionation procedure

The crude methanol extract of papaya leaves (245 g) was dissolved in 1 L of methanol/distilled water (10 : 90 v/v) solution then transferred to a 2 liter-capacity separating funnel to begin the fractionation process *via* partitioning method. The crude dissolved extract was then partitioned with 500 mL of *n*-hexane to separate chlorophyll and non-polar compounds. A total of 7 L of *n*-hexane fraction were then collected and evaporated at 45 °C under reduced pressure using a rotary evaporator. The *n*-hexane fraction was evaporated and then freeze-dried *via* lyophilization giving a total weight of 77 g. The crude extract was further partitioned with ethyl acetate for the isolation of compounds of medium polarity. The crude extract was partitioned with 500 mL of ethyl acetate for twelve runs giving 6 L of ethyl acetate fraction which was subsequently evaporated at 50 °C using a rotary evaporator and then lyophilized to give a solid yellowish-brown ethyl acetate fraction with a net weight of 25 g. As a final fractionation step, the crude extract was partitioned with 500 mL of *n*-butanol for twelve runs to separate the compounds with higher polarity. A total volume of 6 L of the *n*-butanol fraction was collected for further evaporation at 59 °C under reduced pressure using a rotary evaporator then lyophilized producing a total weight of 24 g of a dark brown solid *n*-butanol fraction. The *n*-hexane, ethyl acetate, *n*-butanol fraction and the remaining residue of the crude extract were all freeze dried and stored in the refrigerator for further use.

### 2.5. Metabolic profiling of papaya leaf methanolic extract

LC-MS profiling was performed on methanol extract *C. papaya* according to the method described by Haggag *et al.*^39^ on an Acquity Liquid Chromatography system coupled to a Synapt G2 HDMS quadrupole time-of-flight hybrid mass spectrometer (Waters, Milford, CT, USA). Chromatographic separation was carried out on a BEH C18 column (2.1 × 100 mm, 1.7 μm particle size, Waters, Milford, USA) with a guard column (2.1 × 50 mm, 1.7 μm particle size). A linear binary solvent gradient of 0–100% eluent B over 6 min at a flow rate of 0.3 mL min^−1^, using 0.1% formic acid in water (v/v) as solvent A and acetonitrile as solvent B. The injection volume was 2 μL and the column temperature was 40 °C. LC-MS spectra were viewed using Thermo Xcalibur 2.1 (Thermo Scientific, Germany). MSConvert software was used to convert the raw data into separate positive and negative ionization files. MZmine 2.10 software was used for peak picking, deconvolution, deisotoping, alignment and formula predication. The compounds were identified *via* the Dictionary of Natural Products database.

### 2.6. Anti-SARS-CoV-2 activity

#### 2.6.1. Cells and viruses

Vero-E6 cells were maintained in Dulbecco's Modified Eagle's Medium (DMEM) containing 10% Fetal Bovine Serum (FBS) (invitrogen) and 1% penicillin/streptomycin (pen/strep) antibiotic mixture at 37 °C, 5% CO_2_. All antiviral bioassays were performed against hCoV-19/Egypt/NRC-3/2020 SARS-CoV-2 (NRC-03-nhCoV),^40^ obtained from the virus collections of center of Scientific Excellence for Influenza viruses at National Research Centre, Egypt. To propagate the virus, Vero E6 cells were infected with the virus at a multiplicity of infection (MOI) of 0.1 in infection medium (DMEM containing 2% FBS, 1% pen/strep, and 1% l-1-tosylamido-2-phenylethyl chloromethyl ketone (TPCK)-treated trypsin). Three days post infection, cell culture supernatant was collected and centrifuged for 5 min at 2500 rpm to get rid of cell debris. The supernatant was then aliquoted, and titrated using Tissue Culture Infection Dose 50% (TCID50) End-Point Dilution.^41^

#### 2.6.2. Half maximal cytotoxic concentration (CC_50_) determination

To assess CC_50_ of the tested total extract and relevant fractions, stock solutions were prepared by them in 1× DMEM and serially diluted them with 1× DMEM to prepare the various working concentrations (1 ng mL^−1^ to 1 mg mL^−1^). The CC_50_ of each compound was assayed in Vero-E6 cells by using crystal violet assay as previously described.^42–44^ Briefly, 100 μL of the VERO-E6 cell suspension were distributed into 96-well plates (3 × 10^5^ cells per mL). The seeded plates were then incubated at 37 °C in 5% humidified CO_2_ incubator for 24 h. Cell monolayers were then co-incubated with different concentrations of each in triplicates at 37 °C in 5% humidified CO_2_ incubator. Seventy-two hours later, the media supernatants were discarded, the cell monolayers were washed once with 1× PBS and fixed with 10% formaldehyde for 1 h at room temperature (RT). The plates were further dried and stained at RT with 0.1% crystal violet for 20 min on a bench rocker. The monolayers are then washed, dried, and the crystal violet dye in each well was then dissolved with 200 μL methanol for 20 min on a bench rocker at RT. Eventually, the absorbance was measured at *λ*_max_ 570 nm using the Anthos Zenyth 200rt plate reader (Anthos Labtec Instruments, Heerhugowaard, Netherlands). The cytotoxicity of various concentrations compared to the untreated cells was determined using nonlinear regression analysis by plotting log inhibitor *versus* normalized response.

#### 2.6.3. Inhibitory concentration 50 (IC_50_) determination

The IC_50_ values for the total extract and related fractions were determined as previously described,^45^ with minor modifications. Briefly, the VERO-E6 monolayers in 96-well tissue culture plates were then washed once with 1× PBS. The hCoV-19/Egypt/NRC-3/2020 SARS-CoV-2 (NRC-03-nhCoV, TCID_50_ = 100) was co-incubated with safe serial diluted working concentrations of the tested extract and related fractions at 37 °C for 1 h. The Vero-E6 cells were treated with virus/sample mixtures and kept at 37 °C for 1 h. Untreated/infected cells represented the virus control, however untreated/uninfected cells referred to the cell control. After 72 h of co-incubation at 37 °C in 5% CO_2_ incubator, the cell monolayers were fixed with 100 μL of 10% formaldehyde for 20 min and stained with 0.1% crystal violet “in distilled water” for 15 min at RT. To dissolve crystal violet dye, 100 μL of the absolute methanol were added per well and the optical density of the color is eventually measured at 570 nm using the Anthos Zenyth 200rt plate reader (Anthos Labtec Instruments, Heerhugowaard, Netherlands). The IC_50_ values were calculated using nonlinear regression analysis by plotting log inhibitor *versus* normalized response.

#### 2.6.4. Selectivity index (SI)

The SI is calculated as the ratio of the toxic concentration 50 (CC_50_) of a sample to its effective bioactive concentration 50 (IC_50_) (SI = CC_50_/IC_50_).^46^

### 2.7. Molecular docking

Docking was done as reported earlier.^47^ In brief, ligands 3D structures were downloaded from Pubchem (https://pubchem.ncbi.nlm.nih.gov/) when available or 2D structure is drawn and converted to 3D. Ligands were minimized with 1000 steps of steepest descent algorithm using Avogadro.^48^ Protein targets were retrieved from protein data bank (http://www.rcsb.org/) under the codes 6LU7, 7BV2, 6W4H, 6M0J, 6VW1 and 6WX4. Proteins were prepared by deleting water molecules, adding hydrogens, merging hydrogens and then computing Gasteiger charges using autodock tools. Docking was done using Autodock vina^49^ with a grid box of 25^3^ Å^3^ centred on co-crystalized ligand if available. For 6M0J and 6VW1 where no ligand is available, the grid box was centered on Q493 and E35, respectively. Exhaustiveness of 16 was used for all docking procedures. Co-crystalized ligands were docked when present to validate docking procedure as reported earlier and accepted if RMSD between crystalized and docked ligand is less than 2 Å calculated using DockRMSD server.^50,51^

### 2.8. Molecular dynamics simulation

The chosen complex was then subjected to a 50 ns MD simulation using the AMBER 18 platform.^52^ The receptor structure topology was made using the Leap of Amber19 tools. The addition of Na^+^ ions neutralized the simulation system. The neutralized system was then immersed in the water-molecule-containing TIP3P box. Prior to performing 1000 steps of conjugate gradient, the solvated system underwent 1500 steepest descent method iterations of minimization. With gradual heating from 0 K to 300 K at 1 atm pressure, system heating for 100 ps was accomplished. The system was then allowed to reach equilibrium for 100 ps, allowing the exchange of kinetic and potential energies. A 50 ns simulation production run was completed to assess the dynamics of the complex and confirm the stability of the docked ligand conformation. The SHAKE algorithm was used to impose constraints on hydrogen atoms that were covalently bound. The use of canonical ensemble ensured the periodic boundary conditions in the simulation box. Berendsen was used to maintain a constant temperature in the simulation box. The Ewald summation was used to complete MD simulations, and coordinate files were saved every 0.5 ps. The CPPTRAJ module included in Amber18 was used to analyze the trajectory data.

### 2.9. Binding free energy calculation

Molecular Mechanics-Generalized Born Surface Area (MMGBSA) and Molecular Mechanics Poisson-Boltzmann Surface Area (MMPBSA) embedded in the MMPBSA.py module of AMBER18 were utilized to calculate the binding free energy of the docked complex.^53^ 100 frames were processed from the trajectories in total, and the system's net energy was estimated using the following equation:Δ*G*_binding_ = Δ*G*_complex_ − Δ*G*_receptor_ − Δ*G*_inhibitor_

Each of the forementioned terms requires the calculation of multiple energy components, including van der Waals energy, electrostatic energy, internal energy from molecular mechanics, and polar contribution to solvation energy.

## 3. Results and discussion

### 3.1. Metabolomics profiling of the crude extract of *C. papaya* leaves

The dereplication of secondary metabolites of the crude methanol extract revealed the presence of different classes of phyto-compounds. The positive and negative modes revealed the presence of phenolic, fatty acids, flavonoids, alkaloid, sterol, and triterpenes (Table 1).

**Table 1:** Annotated compounds from the methanolic extract of papaya

*m*/*z*	Retention time (min)	M. wt	Name	Molecular formula	References
739.20	1.79	740.2148625	Clitorin	C_33_H_40_O_19_	54
611.15	8.69	610.1526631	Rutin	C_27_H_30_O_16_	54
595.16	9.05	594.1576881	Nictoflorin	C_27_H_30_O_15_	54
755.20	12.84	756.2090453	Manghaslin	C_33_H_40_O_20_	54
287.05	15.91	286.0475676	Kaempferol	C_15_H_10_O_6_	24
473.36	19.25	472.3547817	2β,3β-Dihydroxy-ursolic acid	C_30_H_48_O_4_	55
487.34	19.74	488.3500919	Acacic acid	C_30_H_48_O_5_	56
457.36	20.64	456.3600077	Oleanolic acid	C_30_H_48_O_3_	57
457.36	23.14	456.3600665	Betulinic acid	C_30_H_48_O_3_	55 and 58
415.38	23.31	414.3819298	β-Sitosterol	C_29_H_50_O	26
479.34	26.48	478.3418738	Carpaine	C_28_H_50_N_2_O_4_	54
479.34	26.48	478.3418738	Pseudocarpaine	C_28_H_50_N_2_O_4_	54
413.36	28.45	412.3550657	Stigmasterol	C_29_H_48_O	37

### 3.2. Anti-SARS-CoV-2 activity

**Fig. 1:**
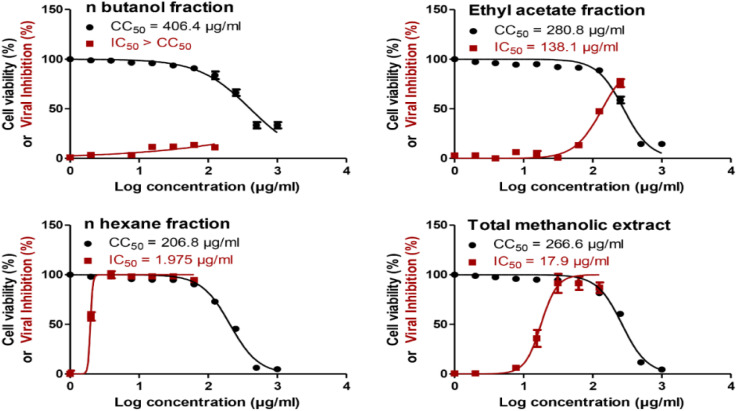
Cytotoxicity (CC_50_) and antiviral activity (IC_50_) for *C. papaya* total methanol extract and its fractions in Vero-E6 cells and against NRC-03-nhCoV in Vero-E6 cells, respectively. The CC_50_ and IC_50_ values were calculated using nonlinear regression analysis of GraphPad Prism software (version 5.01) by plotting log inhibitor *versus* normalized response (variable slope).

The total methanol extract, *n*-hexane, ethyl acetate, and *n*-butanol fractions of papaya leaves were all assessed for their safety against the cells by testing their cytotoxicity on VERO-E6 cells *via* MTT assay. The CC_50_ of the total *n*-butanol extract was the highest with a concentration equal to 406.4 μg mL^−1^. The anti-SARS-CoV-2 activity was assessed by using the crystal violet assay protocol.^44^ The *n*-hexane fraction shows potent antiviral activity against SARS-CoV-2 with an IC_50_ value = 1.98 μg mL^−1^. Although the *n*-butanol extract has the highest CC_50_ against the cells indicating its relative safety compared to the other fractions, the selectivity indices give a more significant explanation to the safety of the tested extracts relative to their antiviral activity. According to the selectivity indices results, the *n*-hexane fraction has the highest selectivity index value = 104.70 with a wide difference than any other extract. This indicated that the *n*-hexane fraction was the most selective to the viral cells without a toxic effect on the other normal cells. The selectivity index was calculated as a ratio that measures the window between cytotoxicity and antiviral activity by dividing the given CC_50_ value into the IC_50_ value (Selectivity Index (SI) = CC_50_/IC_50_). The results of the cytotoxic activity, antiviral activity, and selectivity indices data are presented in Fig. 1 and Table 2.

**Table 2:** The cytotoxic activity, antiviral activity, and selectivity indices of papaya leaves extracts

Extract	CC_50_ (μg ml^−1^)	IC_50_ (μg ml^−1^)	Selectivity index (SI)
*n*-Butanol fraction	406.4	>406.4	<1
Ethyl acetate fraction	280	138.1	2.03
*n*-Hexane fraction	206.8	1.98	104.7
Total methanolic extract	266.6	17.9	14.89

### 3.3. Molecular docking

The genome of the SARS-CoV-2 virus is a large single-stranded RNA which encodes for four main structural proteins that include the nucleocapsid protein (N), membrane protein (M), spike protein (S), and envelope protein (E). In addition, sixteen non-structural proteins (nsp) are also encoded and are responsible for the replication, survival, transcription and pathogenicity.^59^ Our current molecular docking study investigate potential interaction between 13 annotated compounds from the total papaya extract against six SARS-CoV-2 target proteins. These targets include four non-structural proteins which are main protease (Mpro), Papain-like protease (PLpro), RNA dependent RNA polymerase (RdRp) as well as *O*-methyl transferase. Main protease and papain-like proteases are required by the virus to cut the polyprotein chain at specific location to release structural and nonstructural proteins. RdRp is the viral polymerase that is required for viral replication. Furthermore, 2′-*O*-methyltransferase is essential to protect viral RNA from host immune system including the interferon-induced response.^60^ In addition to these non-structural proteins, we also investigated potential recognition of our annotated compounds by receptor binding domain (RBD) of structural S-protein and its human target, angiotensin converting enzyme-2 (ACE-2). Binding of inhibitors to any of the last two targets could interrupt the recognition of S-protein by ACE-2 and hence slow down the infection rate. These targets were investigated by several groups including us and we have validated our docking procedure for all of them earlier.^50,61,62^

A variety of compounds annotated exhibited strong docking scores on the targeted proteins (Table 3). For example, flavonoids showed good docking results with M^pro^ (6LU7). This target protein is responsible for the maturation of replicase and helicase functional enzymes. Therefore, inhibiting M^pro^ is a crucial step for the inhibition of viral replication.^63^ Those flavonoidal compounds include nicotiflorin, which showed the score (−9.3 kca/mol), as well as manghaslin (−8.8 kcal mol^−1^) and rutin (−8.3 kcal mol^−1^) which are all better than the co-crystalized ligand (−7.7 kcal mol^−1^). Nicotiflorin was previously reported to have an affinity on SARS-CoV-2 protease *in silico*.^64^ In fact, those 3 compounds are flavonoid glycosides which might indicate the importance of attached sugars for the recognition *via* hydrogen bonding leading to strong binding. These three compounds also showed best results against 2′-*O*-methyltransferase (6W4H) target protein which is important for viral survival by enhancing viral RNA stability and preventing its degradation by the cellular immunity.^65^ Furthermore, RdRp is considered one of the most important and successful targets against SARS-CoV-2 due to its responsibility for regulating the viral replication and transcription of single-stranded RNA viruses.^65^ For this target, 2β,3β-dihydroxy-ursolic acid showed the best docking score (−8.6 kcal mol^−1^) compared to the remaining tested compounds. In addition, the carpaine alkaloid and rutin flavonoid came next (−8.4 kcal mol^−1^) as inhibitors of RdRp. It worth to mention here that all these compounds are part of the methanolic extract which has shown more than 100-fold selectivity towards SARS-CoV-2 infected cells compared to non-infected Vero-E6 cells (Table 2). Further mechanistic studies are required to confirm the affected targets that are involved in the anti-SARS-CoV-2 activities.

**Table 3:** Docking scores (kcal mol^−1^) for annotated compounds with different SARS-CoV-2 targets

Compound	Docking scores (kcal mol^−1^)					
	Mpro	RdRp	O-ME transferase	ACE-2	RBD-S-protein	PLpro
Clitorin	−7.8	−8.1	−8.3	−5.4	−6.8	−6.8
Rutin	−8.3	−8.4	−8.7	−5.6	−6.6	−7
Nicotiflorin	−9.3	−8.2	−9.3	−5.9	−7.4	−6.9
Manghaslin	−8.8	−8.2	−8.7	−5.7	−7	−6.8
Kaempferol	−7.9	−7.5	−8	−7	−6	−6.8
2β,3β-Dihydroxy-ursolic acid	−7.2	−8.6	−8.1	−6	−6.8	−7
Acacic acid	−7.2	−7.2	−8	−5.8	−6.6	−6.9
Oleanolic acid	−7.3	−7.5	−8	−5.8	−6.5	−6.8
Betulinic acid	−7.1	−7.4	−7.8	−6	−6.6	−7.2
β-Sitosterol	−7.2	−6.3	−7.6	−5.2	−6.1	−6.8
Carpaine	−7.8	−8.4	−8.5	−6.3	−7	−7.4
Pseudocarpaine	−7.7	−8	−8.2	−6.1	−7.4	−7.8
Stigmasterol	−7.2	−6.3	−7.7	−5.8	−7	−6.7
Co-crystallization ligand	−7.7	−6.8	−8.2	—	—	−6.8

**Fig. 2:**
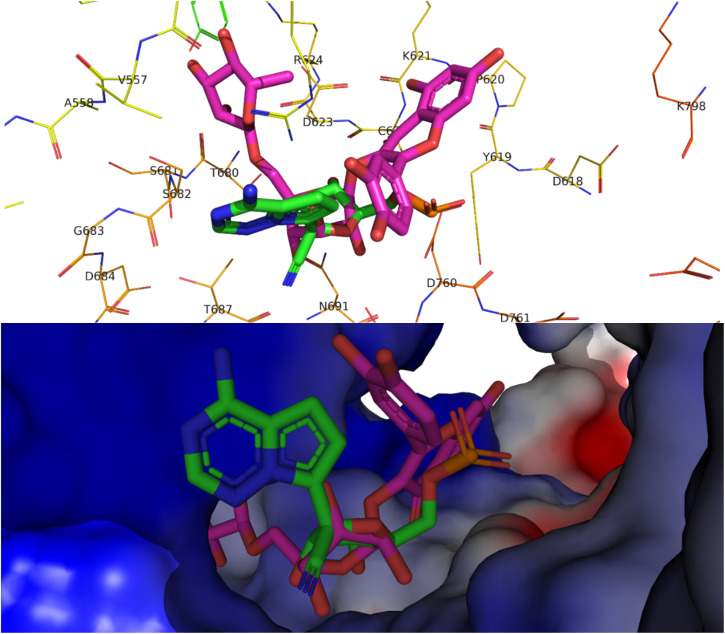
Top, binding pose of rutin (pink) in the active site of SARS-CoV-2 RdRp (7BV2) overlapped with Remdesivir (green). Bottom, surface representation of rutin (pink) in the active site of SARS-CoV-2 RdRp (7BV2) overlapped with Remdesivir (green).

Alkaloids like pseudocarpaine and carpaine showed significant binding scores on all targeted proteins except ACE-2 especially pseudocarpaine which exhibited the best binding score (−7.4 kcal mol^−1^) on RBD-spike protein (6M0J) which is responsible for the viral attachment and entering the host cell. Therefore, binding to the spike protein could alter the process of viral attachment and prevent the infection process.^65^ Those alkaloids also significantly showed the best binding scores among all annotated compounds on PL^pro^ (6WX4) enzyme which could help in the prevention of the viral polyproteins processing responsible for the viral spread.^66^ In addition, terpenes such as betulinic acid, acacic acid and oleanolic acid showed better fitting scores on RNA-dependent RNA polymerase (7BV2) and papain-like protease (PL^pro^) (6WX4) than the co-crystallized ligands. Sterols like β-sitosterol and stigmatserol exhibited a better fitting score than the Co-Crystallized ligand on PL^pro^ (6WX4) and RBD-Spike protein (6M0J) respectively. Moreover, some annotated flavonoids such as manghaslin, clitorin, rutin and kaempferol showed moderate to high fitting score on the targeted proteins. The docking score results of the annotated compounds are presented in Table 3. Furthermore, a binding pose of rutin on the active site of SARS-CoV-2 RdRp (7BV2) is demonstrated in Fig. 2.

**Fig. 3:**
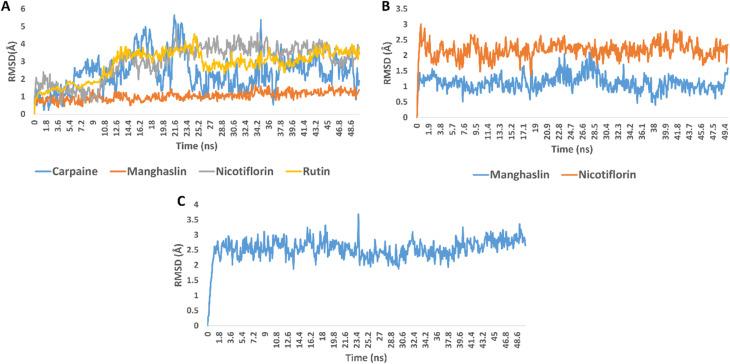
RMSDs of carpaine, manghaslin, nicotiflorin, and rutin inside OMT's active site (A); manghaslin and nicotiflorin inside Mpro's active site (B); dihydroxyursolic acid inside RdRp's active site (C) over 50 ns-long MDS runs.

In order to validate the docking results, we subjected the structures of the best scoring compounds to 50 ns-long MDS in addition to MM-PBSA calculation of their binding energy. As shown in Fig. 3, all compounds' structures showed comparable binding stability inside the three proteins' active sites. Regarding OMT, carpaine, nicotiflorin, and rutin were significantly unstable inside the enzyme's active site in comparison with manghalsin (Fig. 3A), where they were highly fluctuating over the course of simulations with high average RMSDs (4.43 Å, 3.89 Å, and 3.45 Å, respectively). In case of manghalsin and, nicotiflorin with Mpro, both structures showed acceptable binding stability inside the enzyme's active site with average RMSDs of 1.42 Å and 2.21 Å, respectively (Fig. 3B). Similarly, dihydroxyursolic acid achieved good binding stability inside RdRp's active site with an average RMSD of 2.53 Å (Fig. 3C).

Moreover, we estimated the binding free energy of each compound inside the corresponding protein targets using the MM-PBSA method.^67^ The obtained results were in good accordance with that stability profile of each compound. Carpaine and nicotiflorin with OMT were the lowest scoring compounds in terms of binding free energy (Table 4).

**Table 4:** Binding free energies for the 7 selected compounds in complex with OMT, Mpro, and RdRp

Energy component	Carpaine-OMT	Manghaslin-OMT	Nicotiflorin-OMT	Rutin-OMT	Manghaslin-Mpro	Nicotiflorin-Mpro	Dihydroxyursolic acid-RdRp
DELTA G gas	−27.5602	−54.9876	−21.8203	−38.1047	−48.4837	−39.3409	−36.8341
DELTA G solv	9.5982	16.7483	11.2943	13.6452	16.3827	14.8213	15.3823
DELTA total	−17.962	−38.2393	−10.526	−24.4595	−32.101	−24.5196	−21.4518

The *in vitro* results of this study (Table 2) revealed that the *n*-hexane fraction had the highest anti-SARS-CoV-2 activity with a significant margin compared to the total methanol extract and the other fractions with an IC_50_ value = 1.98 μg mL^−1^ and significantly better selectivity index = 104.7. Therefore, the *n*-hexane fraction is considered the most effective fraction against SARS-CoV-2 compared to the other fractions and the total extract. According to molecular docking results, it can be deduced that the presence of terpenoids such as 2β,3β-dihydroxy-ursolic acid which showed the best binding score on RdRp and betulinic acid, acacic acid and oleanolic acid which demonstrated good fitting scores on the same protein could help in inhibiting viral replication process. The annotated Sterols had high to moderate binding scores on the targeted PL^pro^ and RBD-spike protein which may contribute to the prevention of the viral attachment to the host cell and the viral spread. This could relate to the high anti-SARS-CoV-2 activity of the *n*-hexane fraction. In addition, alkaloids such as pseudocarpaine and carpaine showed a good binding on most of the targeted proteins. Studies displayed the presence of such alkaloids in many fractions of papaya leaves including the hexane fraction which explains their role in altering the viral replication and spreading process.^7,56,68^ Other studies suggest the presence of traces of flavonoids in the hexane fraction.^68–70^ All these findings indicate that all the compounds that possess high scores on the targeted proteins may contribute to the high *in vitro* activity of the hexane fraction through synergism. However, further phytochemical and biological investigations are needed to identify more compounds that could have a role in the explanation of the prominent activity of the hexane fraction and the other fractions of papaya leaves against SARS-CoV-2.

## 4. Conclusion

The previously reported antiviral activities of *C. papaya* led us to investigate its activity against SARS-CoV-2. The total methanol extract, *n*-hexane, ethyl acetate, and *n*-butanol fractions of papaya leaves were subjected to anti-SARS-CoV-2 activity and cytotoxicity assays. The results showed that the *n*-hexane fraction had the highest anti-SARS-CoV-2 activity besides having the highest selectivity index. This result assumes that the high activity of the *n*-hexane fraction against SARS-CoV-2 may be due to the presence of some classes of compounds that existed in papaya such as fatty acids, sterols, and triterpenes which were previously reported to have a wide range against different classes of viruses including some compounds that have a reported anti-SARS-CoV-2 activity. In addition, the molecular docking study together with the molecular dynamics simulations and MM-PBSA calculation demonstrated the moderate to high fitting potential of the annotated compounds with the targeted SARS-CoV-2 proteins. The annotated compounds belong to different classes that include sterols and triterpenes, flavonoids, and alkaloids. These findings open the door to further investigations that concern the compounds responsible for the activity of *C. papaya* leaves against SARS-CoV-2 and the *n*-hexane extract in particular.

## Author contributions

Amr Adel: conceptualization, methodology, writing and editing. Mohamed S. Elnaggar: supervision, reviewing and validation. Amgad Albohy: methodology, investigation and editing. Ahmed A. Elrashedy: methodology and investigation. Ahmed Mostafa: Methodology, editing and funding acquisition. Omnia Kutkat: methodology and investigation. Usama Ramadan Abdelmohsen: methodology and investigation. Eman Alsayed: supervision, reviewing and validation. Mohamed A. Rabeh: supervision and reviewing. All authors have read and approved the final manuscript.

## Conflicts of interest

The authors declare that they have no conflicts of interests.

## Supplementary Material
